# Cannabis use in the UK: a quantitative comparison of individual differences in medical and recreational cannabis users

**DOI:** 10.3389/fpsyg.2023.1279123

**Published:** 2024-01-08

**Authors:** Beata Ciesluk, Simon Erridge, Mikael H. Sodergren, Lucy J. Troup

**Affiliations:** ^1^Division of Psychology, School of Education and Social Sciences, University of the West of Scotland, Paisley, United Kingdom; ^**2**^Medical Cannabis Research Group, Imperial College London, London, United Kingdom; ^3^Sapphire Medical Clinics, London, United Kingdom; ^4^Curaleaf International, London, United Kingdom

**Keywords:** cannabis, cannabinoid, medical cannabis, recreational drug use, anxiety, depression

## Abstract

There is a paucity of research, especially in the UK, that investigates individual differences in both medical and recreational cannabis users. A cross-sectional survey study design was used to assess recreational cannabis users and medical cannabis users currently living in the UK. Recreational cannabis users were invited to take part via social media. Medical cannabis users were recruited from Sapphire Medical Clinics, London, UK, which provides treatment with prescribed cannabis-based medicinal products. Demographic data and cannabis use frequency, as well as post-traumatic stress disorder symptoms (PCL-5), depression symptoms (Centre for Epidemiological Studies Depression Scale), trait and state anxiety (State-Trait Anxiety Inventory), and cannabis use motives [Comprehensive Marijuana Motives Questionnaire (CMMQ)] were collected. The Chi-square and independent-sample *t*-tests were used for the comparison of categorical variables and normally distributed continuous variables. Data were analyzed using analyses of variance (ANOVAs) and *t*-tests. Statistical significance was considered where the value of *p* was <0.05. The survey was completed by 161 participants. Medical cannabis users were older, consumed cannabis more often, had a higher “Sleep” motive on the CMMQ, and had a higher prevalence in self-reporting current diagnoses of neurological problems, mood disorders, and anxiety disorders (*p* < 0.05). Recreational cannabis users had higher scores on several motives for use (e.g., “Enjoyment,” “Coping,” “Experimentation,” “Boredom,” and “Celebration”) and higher state anxiety scores (*p* < 0.05). The most common motives for cannabis use in both groups were “Enjoyment,” “Low Risk,” and “Sleep.” There were no differences between groups in gender, “Low-Risk” motive, post-traumatic stress disorder symptoms, depression scores, trait anxiety scores, self-reported prevalence of substance use-related disorders, and past consumption of alcohol, tobacco, or caffeine (*p* > 0.05). The current study not only demonstrates a difference in age and motivations for cannabis consumption between recreational and medical cannabis users but also shows areas of potential overlap, including mental health outcomes, past substance use, and gender. These UK-specific findings indicate that recreational cannabis users experience higher state anxiety, highlighting the need for further evaluation of potential anxiogenic/anxiolytic properties of cannabis. These findings demonstrate the importance of researching individual differences in cannabis users and hold significant implications for future research, clinical practice, and legislation.

## 1 Introduction

There is a growing consumption of cannabis for both recreational and medical reasons globally (Azofeifa et al., 2016; Hasin and Walsh, 2021; Manthey et al., 2021; Kalayasiri and Boonthae, 2023). In the UK, cannabis is the most commonly used illicit drug, with 7.4% of adults between 16- to 59-years-old reporting having used the drug within the past year (Office for National Statistics, 2022). The introduction of legislative reform in the UK in 2018 has also facilitated the prescription of cannabis-based medicinal products (CBMPs) under medical supervision (Torjesen, 2018). Despite legislative change, there is limited understanding of the differences between medical cannabis users (MCUs) and recreational cannabis users (RCUs) (Pacula et al., 2016). This is especially true in the UK, where there has been a paucity of research compared to the USA and Canada (Schlag et al., 2020). This lack of research has been implicated as a barrier to the implementation of CBMPs in the UK in part due to perceived stigma by patients prescribed CBMPs (Troup et al., 2022), potentially leading to hesitancy to prescribe CBMPs (Morris, 2020; Nutt et al., 2020; Schlag et al., 2020).

There is also a relative deficit in high-quality evidence on the individual differences between MCUs and RCUs. Existing comparisons derive mainly from large-scale population studies in the USA. These studies have illustrated that, compared to RCUs, MCUs are typically older (Choi et al., 2017; Camsari et al., 2019), more likely to report daily consumption (Lin et al., 2016; Choi et al., 2017), have worse overall physical health (Roy-Byrne et al., 2015; Lin et al., 2016), and are more likely to report psychiatric comorbidities, including post-traumatic stress disorder (PTSD), depression, and anxiety (Choi et al., 2017). Research has shown that MCUs are less likely to use illicit drugs (Lin et al., 2016) compared to RCUs, who appear more likely to be polydrug users. However, these findings are likely reflective of the fact that psychiatric conditions are one of the more common classes of disorders for which CBMPs are prescribed rather than medical cannabis use, leading to a higher incidence of psychiatric conditions (Zongo et al., 2022; Olsson et al., 2023). Yet, considering the potential for cannabinoids to induce positive or negative effects on mental health outcomes according to relevant doses consumed, it is important to continue to examine the differences between RCUs and MCUs, considering the underlying differences in their pattern of consumption and underlying traits driving cannabis consumption (Sharpe et al., 2020). This lack of understanding of the similarities and differences between these two groups makes it difficult to legislate in respect of both the medical application and potential risk of recreational or illicit cannabis use.

Prior attempts to evaluate the differences between MCUs and RCUs have focused on recruiting from a general population rather than targeted assessment of individuals with a confirmed prescription for CBMPs (Roy-Byrne et al., 2015; Choi et al., 2017; Camsari et al., 2019). There may be a social desirability bias in reporting cannabis consumption to be medical in nature. This may, therefore, lead to inappropriately including RCUs within a sample of MCUs, limiting the conclusions that can be drawn from those data sets. Where prior comparisons have been conducted between MCUs and RCUs, they have not explored the reasons why differences exist. Different motives for cannabis use have been associated with cannabis use problems (Bresin and Mekawi, 2019), cannabis use patterns (Bonn-Miller et al., 2007; Casajuana-Kögel et al., 2021), and psychiatric disorders (Buckner et al., 2012; Metrik et al., 2016). Examining the motives for cannabis use could provide insights into differences between MCUs and RCUs. For example, previous research has reported that MCUs display an increased frequency of cannabis use and higher psychiatric symptoms (Choi et al., 2017; Turna et al., 2020), which is likely reflective of the frequency required for the relief of the associated symptoms for which CBMPs are used (Bonn-Miller et al., 2014). RCUs tend to use cannabis for enjoyment (Zvolensky et al., 2007) or to experiment and socialize (Bonn-Miller et al., 2014), which could be associated with the heightened polydrug use in this cohort (Lin et al., 2016). Therefore, previous research (Lin et al., 2016; Choi et al., 2017; Turna et al., 2020) has omitted an important factor when examining the differences between RCUs and MCUs. Whilst one significant difference between the two groups is that MCUs are assumed to be primarily using cannabis to alleviate symptoms of an underlying condition, it is also possible that RCUs are also accessing cannabis to self-medicate to varying levels. An example of this is evident in attention-deficit/hyperactivity disorder where some individuals report that their cannabis use is associated with improvements in hyperactivity and impulsivity (Mitchell et al., 2016). To provide a more in-depth understanding of the differences between the groups, improved research, which includes an investigation of motives for cannabis use, is required.

The primary aim of the current study was to compare individual differences in RCUs and MCUs in the UK to investigate potential motivations for use that may reflect overlap or divergence between cannabis users from two distinct groups. Specifically, the study aimed to analyze the differences between RCUs and MCUs with respect to their mental health and their motives for cannabis use, as well as individual differences, including age and cannabis use frequency, as well as caffeine, alcohol, and tobacco use.

## 2 Method

### 2.1 Participants

Cannabis users currently living in the UK were invited to participate in the study by recruiting via either social media for RCUs or Sapphire Medical Clinics for MCUs. Sapphire Medical Clinics was the first medical cannabis clinic in the UK to be registered with the regulatory authorities. It is currently the largest clinic with a geographically diverse population spanning all four nations within the UK and the crown dependencies. The invitation to participate included an online link to the survey, which was delivered via Question-Pro (Survey Analytics LLC, Oregon, United States).

To ensure that RCUs and MCUs are separated into two distinct groups, two individual links to the online survey were made. MCUs invited to take part in the survey were recruited from Sapphire Medical Clinics who had consented to being contacted regarding research and had a minimum of two appointments at the clinic and a minimum of one prescription of a CBMP in the past 3 months (*n* = 3,616). A total of 296 participants responded to the survey. 151 RCUs responded to the advertisement placed online (Facebook, Instagram, Twitter, and Reddit). Overall, 70 (46.36%) participants from this sample were excluded because they failed to complete the survey, and 1 (0.66%) participant was excluded as they reported never consuming cannabis. A total of 145 (4.01%) participants responded to the invitations from the Sapphire Medical Clinics. Overall, 64 (44.44%) participants were excluded because they failed to complete the survey, leaving a total sample of 81 (55.86%) MCUs.

Participants provided informed consent, and all research was conducted in line with the principles outlined in the Declaration of Helsinki (World Medical Association, 2013). Ethical approval for the study was granted by the ethics committee of the School of Education and Social Sciences of The University of the West of Scotland (approval number: 2022-18118-15377).

### 2.2 Design

The current study used a cross-sectional questionnaire-based design. Data were collected online using Question-Pro (Survey Analytics LLC, Oregon, United States) between 14 June and 14 July 2022.

### 2.3 Measures and apparatus

The online survey was devised by a multi-disciplinary team of researchers, including academic physicians, a clinical cognitive neuroscientist, and a Community Link Worker with expertise in drug and alcohol support. The questionnaire included questions on demographics (age, gender, nationality, other substance use, and psychological health) and measures assessing cannabis use motives, depression, anxiety, and PTSD symptoms.

#### 2.3.1 Comprehensive Marijuana Motives Questionnaire

To measure cannabis use motives, the Comprehensive Marijuana Motives Questionnaire (CMMQ) was used (Lee et al., 2007, 2009). CMMQ is a 36-item measure which prompts participants to indicate the frequency with which they use cannabis for 12 distinct reasons (*Enjoyment, Conformity, Coping, Experimentation, Boredom, Alcohol, Celebration, Altered Perception, Social Anxiety, Low Risk, Sleep, and Availability*), using a scale of 1 (almost never or never) to 5 (almost always or always). Each of the 12 distinct reasons has a composite score of 1–15. Higher scores indicate a greater value placed on that motive for using cannabis. Previous research conducted in the USA supports the utility of the CMMQ among RCUs (Blevins et al., 2016) and MCUs (Bohnert et al., 2018).

#### 2.3.2 The PTSD checklist

To assess participants’ PTSD symptoms, the PTSD Checklist (PCL-5) was used (Blevins et al., 2015). PCL-5 is a 20-item measure, with four clusters of symptoms that correspond to the DSM-5 as follows: Cluster B (intrusion symptoms), Cluster C (avoidance of stimuli), Cluster D (negative alterations in mood or cognition), and Cluster E (alterations in arousal and reactivity). Responses are scored on a scale of 0 (not at all) to 4 (extremely), with total scores ranging from 0 to 80. Scores over 33 are considered as representing a probable diagnosis of PTSD. Participants were asked to answer PCL-5 items based on their most traumatic event. PCL-5 has good test–retest reliability and convergent and discriminant validity (Blevins et al., 2015).

#### 2.3.3 Centre for Epidemiological Studies Depression Scale

To assess participants’ depressive symptoms, the Centre for Epidemiological Studies Depression Scale (CES-D) was used (Radloff, 1977). The CES-D contains 20 items and includes six components (depressed mood, feelings of guilt and worthlessness, feelings of helplessness, psychomotor retardation, loss of appetite, and sleep disturbance). Participants indicated the frequency of the symptoms in the last week, using a scale of (0) “rarely or none of the time” (<1 day) to (4) “most or all of the time” (5–7 days). The total range of scores is from 0 to 60, with higher values representing greater severity of symptoms. Early validation studies indicate that the CES-D has high internal consistency, acceptable test–retest reliability, and good construct validity in both clinical and community samples (Radloff, 1977).

#### 2.3.4 State–Trait Anxiety Inventory

To assess participants’ anxiety, the State–Trait Anxiety Inventory (STAI) was used (Spielberger et al., 1983). The STAI is comprised of separate State and Trait scales. Each scale has 20 four-point items. The State scale prompted participants to rate the intensity of anxiety symptoms experienced at that moment (‘not at all’ to ‘very much so’). The Trait scale generally assessed participants’ anxiety in terms of intensity (‘almost never’ to ‘almost always’). Scores over 35 on both portions of the STAI are considered high. Early validation studies indicate that STAI has good construct validity (Smeets et al., 1997), discriminant and convergent validity (Spielberger et al., 1983), and test–retest reliability (Rule and Traver, 1983).

### 2.4 Procedure

Prior to the self-reported online survey, participants were provided with an information sheet with the aim and purpose of the study. The survey began with demographic questions about age, gender, ethnicity, consumption of caffeine, alcohol, nicotine, and cannabis, as well as questions regarding current psychological diagnoses. Following, participants answered the CMMQ (Lee et al., 2007, 2009), the CES-D (Radloff, 1977), the STAI (Spielberger et al., 1983), and the PCL-5 (Blevins et al., 2015).

### 2.5 Statistical analysis

Data were curated in Excel (Microsoft 365, Microsoft, WA, USA), and appropriate statistical tests of significance were used to evaluate differences between groups. The Chi-square and independent-sample *t*-tests were used for comparison of categorical variables and normally distributed continuous variables. Differences in motives and mental health outcomes between the two cohorts were analyzed using analyses of variance (ANOVAs) and planned *t*-tests. *p*-values for all statistical analyses were considered significant below 0.05. All data were prepared and analyzed using Jeffreys’s Amazing Statistics Program [JASP Team (2023); JASP (Version 0.16.3) Microsoft Windows 10, Microsoft, WA, USA].

## 3 Results

### 3.1 Demographics

Table 1 displays the complete demographic and clinical characteristics of RCUs and MCUs.

**Table 1:** Demographic and clinical characteristics in RCUs and MCUs.

Baseline characteristic	RCUs	RCUs	MCUs	MCUs	Significant test statistic and value of *p*
	*n*	%	*n*	%	
Gender					
Women	27	33.75	26	32.10	
Men	52	56.00	50	61.70	
Other	1	1.25	4	4.90	
Prefer not to say	0	0.00	1	1.20	
Ethnicity					
White	68	85.00	75	92.60	
Hispanic/Latino/Spanish Origin	1	1.25	0	0,00	
Black/African American	2	2.50	1	1.23	
Asian	2	2.50	1	1.23	
Middle Eastern or North African	1	1.25	1	1.23	
Other race, ethnicity, or origin	6	7.50	3	4.00	
Age range (Years)					
18–24	16	20.00	9	11.11	
25–34	46	57.50	22	27.16	*χ*^2^ = 15.19, *p* < 0.001
35–44	11	13.75	26	32.10	*χ*^2^ = 7.66, *p* = 0.006
45–55	5	6.25	19	23.45	*χ*^2^ = 9.39, *p* = 0.002
56–70	2	2.50	5	6.17	
Substance use (Past 24 h)					
Caffeine	62	77.50	58	72.50	
Alcohol	24	30.00	20	25.00	
Tobacco	40	50.00	32	40.00	
Cannabis	53	66.25	71	88.75	*χ*^2^ = 10.42, *p* < 0.001
Substance use (Past 8 h)					
Caffeine	54	67.50	49	61.25	
Alcohol	10	12.50	5	6.25	
Tobacco	36	45.00	31	38.75	
Cannabis	32	40.00	49	61.25	*χ*^2^ = 7.60, *p* = 0.006
Current psychological diagnoses					
Neurological problem	11	13.75	24	29.63	*t* = 2.47, *p* = 0.014
Mood disorder	8	10.00	27	33.30	*t* = 3.72, *p* < 0.001
Anxiety disorder	17	21.25	38	46.91	*t* = 3.54, *p* < 0.001
Substance-related disorder	1	1.25	2	2.47	
Any other psychological disorder	10	12.50	15	18.51	

Significant group differences at *p* = 0.05 are Shown. RCU *N* = 80, MCU *N* = 81, (total sample = 161). RCU, Recreational Cannabis Users; MCU, Medical Cannabis Users.

#### 3.1.1 Gender and ethnicity

There were no significant differences in gender between groups. In both, there was a higher frequency percentage of men, with 56.00% of men (*n* = 52) belonging the RCU group and 61.70% of men (*n* = 50) belonging to the MCU group. There were no significant differences in ethnicity, with both groups displaying a higher frequency percentage of self-reporting as white.

#### 3.1.2 Age range

**Figure 1:**
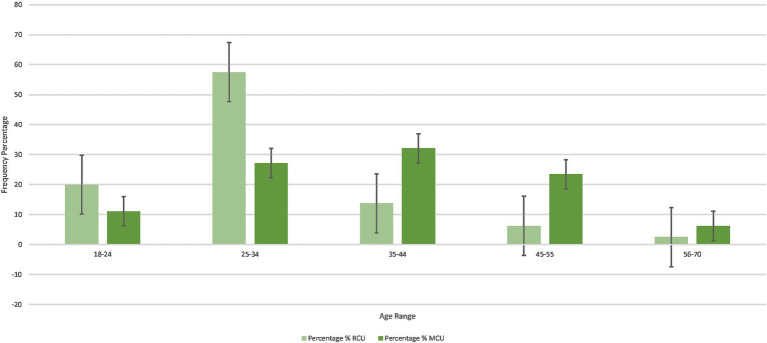
Frequency percentage of age range in RCUs and MCUs.

There were significant differences between the 25–34, 35–44, and 45–55 year age groups. RCUs had a higher frequency of reporting being in the 25–34 age group (*n* = 46; 57.50%) than MCUs (*n* = 22; 27.16%; *p* < 0.001), and MCUs had a higher frequency of reporting being in the 35–44 (*n* = 26; 32.10%) and 45–55 age groups (*n* = 19; 23.45%) than RCUs (*n* = 11; 13.75%; *p* = 0.006) (*n* = 5; 6.25%; *p* = 0.002). The differences in age range groups between RCUs and MCUs are further displayed in Figure 1.

To further investigate the differences between the age ranges in the two groups, the age range groups were scored on a 1–5 scale (1 being the 18–24 age group, 5 being the 56–70 age group). The independent sample *t*-test showed a significant overall difference between the RCU and MCU age range groups, *t*(159) = 4.610, *p* < 0.001. This indicates that MCUs had a higher overall score on age range groups (*M* = 2.86, *SD* = 1.09) than RCUs (*M* = 2.13, *SD* = 0.9), exemplifying that MCUs are, on average, older than RCUs.

#### 3.1.3 Substance Use

**Figure 2:**
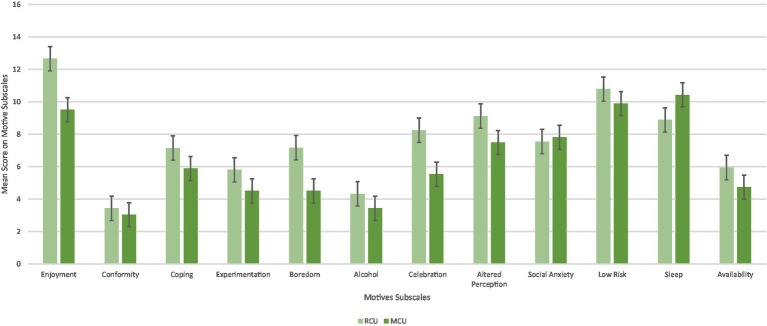
Frequency percentage of substance use prior to completing the survey.

Considering substance use prior to completing the survey, there were only significant differences for cannabis use (*p* = 0.006) (see Figure 2; Table 1), with MCUs presenting a higher frequency of cannabis use 24 h prior to taking the survey (*n* = 71; 88.75%) than RCUs (*n* = 53; 66.25%; *p* < 0.001) and 8 h prior to completing the survey (*n* = 49; 61.25%; *p* = 0.006) than RCUs (*n* = 32; 40%; *p* = 0.006). There were no significant differences in caffeine, alcohol, and tobacco use between the two groups (*p* > 0.050).

#### 3.1.4 Current psychological diagnoses

**Figure 3:**
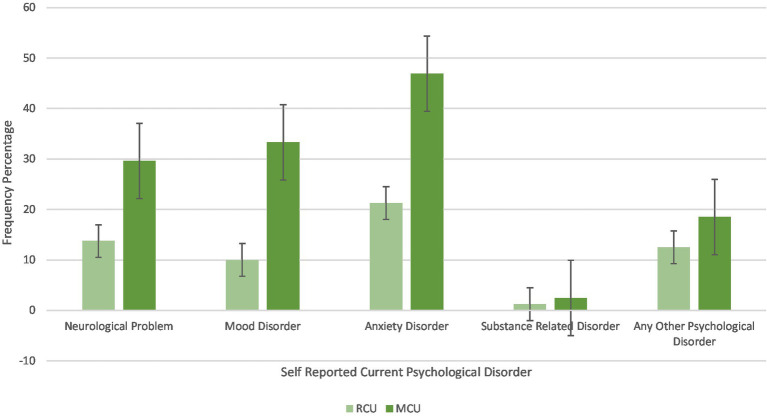
Differences in percentage frequency of self-reported current psychological diagnoses between RCU and MCU.

Regarding self-reported current psychological diagnoses, MCUs show a greater prevalence of current neurological problems (*p* = 0.014), mood disorders (*p* < 0.001), and anxiety disorders (*p* < 0.001). No differences between the two groups in substance use-related disorders and other psychological disorders were found (*p* > 0.050) (see Figure 3; Table 1).

### 3.2 Mental health

The differences between the mental health measure scores of the RCU and MCU age groups were analysed using a mixed-design (2 × 4) ANOVA, with within-subject factors of mental health measure scores (PCL-5, CES-D, STAI_S, and STAI_T) and between-subject factors of the cannabis user group (RCU and MCU) (see Table 2). The descriptive statistics for each measure and each group are reported in Table 3. Of note, two participants were removed from the MCU group for this analysis as they failed to complete the measures.

**Table 2:** Results of 2 × 4 ANOVA on the differences between RCU and MCU on mental health measures scores.

	ANOVA			
Factors	*F*	df	*p*	*η* ^2^
Mental health	457.4	1.93	0.001	0.32
Cannabis user	0.10	1	0.75	3.38
Mental Health*Cannabis User Group	5.07	1.93	0.007	0.004

RCU, Recreational Cannabis User; MCU, Medical Cannabis Users.

**Table 3:** Descriptive statistics for mental health measures scores per RCU and MCU group, results from *t*-test and *χ*^2^ analyses.

Mental health measure	RCUs		MCUs		*t*-Test			*χ* ^2^	
	*M*	*SD*	*M*	*SD*	*T*	*p*	Cohen’s	*χ* ^2^	*p*
Depression^a^	15.89	10.76	17.32	13.03	0.95	0.34	0.15	42.24	0.504
PTSD^b^	20.66	18.35	23.96	20.54	1.20	0.23	1.18	65.07	0.274
Anxiety Trait^c^	40.58	11.15	41.38	14.77	0.60	0.57	0.09	47.00	0.469
Anxiety State^d^	40.54	5.40	37.61	14.75	1.60	0.09	−0.26	91.42	0.001

^a^Center for Epidemiological Studies-Depression Scale (CES-D).

^b^The PTSD Checklist (PCL-5).

^c^Trait portion of State–Trait Anxiety Inventory (STAI).

^d^State portion of State–Trait Anxiety Inventory (STAI).

RCU, Recreational Cannabis Users; MCU, Medical Cannabis Users.

Mauchly’s test of sphericity indicated that the assumption of sphericity had been violated [*χ*^2^(5) = 0.46, *p* < 0.001]; therefore, degrees of freedom were corrected using Greenhouse–Geisser estimates of sphericity (*ε* = 0.64).

**Figure 4:**
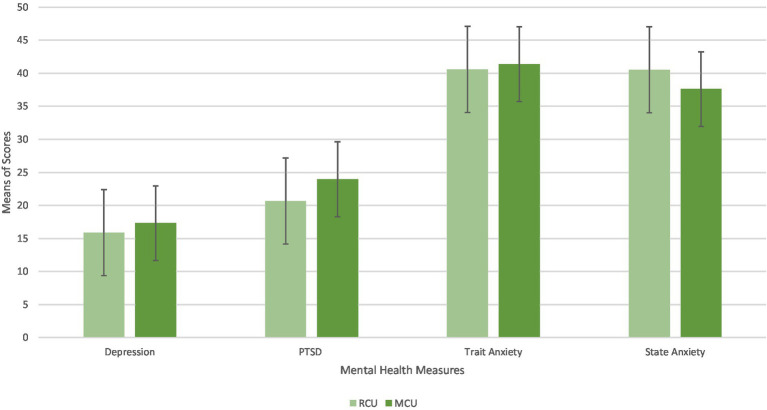
Differences in mean scores on mental health measures between RCU and MCU.

Using the Greenhouse–Geisser correction, there was a main effect of mental health (*Depression, PTSD, Trait Anxiety,* and *State Anxiety*), *F*(1.93, 303.09) = 457.4, *p* < 0.001, *η*^2^ = 0.32. Using the Greenhouse–Geisser correction, there was a main effect of Mental Effect*Cannabis User interaction, *F*(1.93, 303.09) = 5.07, *p* = 0.007, *η*^2^ = 0.004. This interaction is illustrated in Figure 4. There was no significant main effect of the cannabis user group.

**Figure 5:**
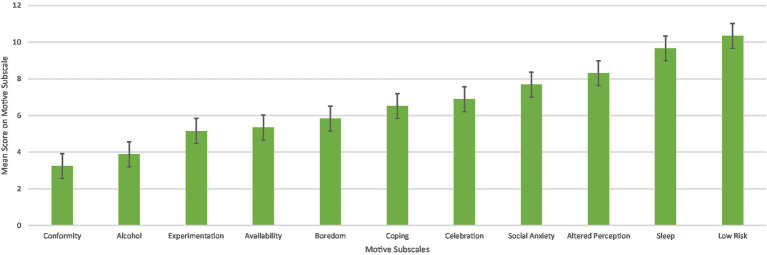
Total sample mean scores on motives subscales.

Planned independent-sample *t*-tests were conducted to examine the significant main effect of Mental Effect*Cannabis User interaction. As observed in Figure 5, RCUs scored lower on all the mental health measures except from *State Anxiety* scores (*p* < 0.001). As shown in Table 3, there were no significant differences between any of the measures on the cannabis user group; however, the difference between RCU *State Anxiety* scores (*M* = 40.54, *SD* = 5.4) and MCU *State Anxiety* scores (*M* = 37.61, *SD* = 14.75, *p* = 0.09) approached significance.

Furthermore, the chi-squared tests were conducted to analyze whether each mental health measure differed between the two groups. As shown in Table 3, there was only a significant difference between RCUs and MCUs on the State Anxiety measure *χ*^2^(41, 159) = 91.418, *p* < 0.001, demonstrating a difference between higher RCU *State Anxiety* scores and lower MCU *State Anxiety* scores.

### 3.3 Motives

The differences in motives for cannabis use between RCUs and MCUs were assessed by the CMMQ, which has 12 subscales (*Enjoyment, Conformity, Coping, Experimentation, Boredom, Alcohol, Celebration, Altered Perception, Social Anxiety, Low Risk, Sleep*, and *Availability*). Descriptive statistics for each subscale and group scores are displayed in Table 4.

**Table 4:** Descriptive statistics and *t*-test results for differences in motive subscales between RCUs and MCUs.

Motive subscales	Total sample		RCUs		MCUs		*T*	*p*	Cohens
	*M*	*SD*	*M*	*SD*	*M*	*SD*			
Enjoyment	11.08	3.54	12.66	2.51	9.52	3.72	−6.284	<0.001	−0.990
Conformity	3.24	1.00	3.44	1.37	3.04	0.25	−2.594	0.010	−0.409
Coping	6.52	3.53	7.15	3.68	5.89	3.28	−2.294	0.023	−0.362
Experimentation	5.15	2.96	5.81	3.19	4.51	2.58	−2.859	0.005	−0.451
Boredom	5.83	3.38	7.18	3.68	4.51	2.41	−5.448	<0.001	−0.859
Alcohol	3.88	2.03	4.32	2.32	3.43	1.60	−2.852	0.005	−0.449
Celebration	6.88	4.01	8.25	3.96	5.53	3.61	−4.556	<0.001	−0.718
Altered perception	8.30	4.40	9.13	4.35	7.49	4.33	−2.384	0.018	−0.376
Social anxiety	7.68	4.42	7.55	4.48	7.82	4.38	0.379	0.705	0.060
Low risk	10.34	3.92	10.79	3.76	9.89	4.05	−1.458	0.147	−0.230
Sleep	9.67	4.14	8.89	4.42	10.43	3.72	2.401	0.017	0.379
Availability	5.33	2.73	5.94	3.02	4.74	2.29	−2.840	0.005	−0.448

RCU, Recreational Cannabis Users; MCU, Medical Cannabis Users.

The differences in motives between MCUs and RCUs were analyzed using a mixed-design (2 × 12) ANOVA (see Table 5) with within-subject factors of motives subscales (*Enjoyment, Conformity, Coping, Experimentation, Boredom, Alcohol, Celebration, Altered Perception, Social Anxiety, Low Risk, Sleep, and Availability*) and between-subject factors of cannabis user group (RCUs and MCUs). Mauchly’s test of sphericity indicated that the assumption of sphericity had been violated [*χ*^2^(65) = 0.084, *p* < 0.001]; therefore, degrees of freedom were corrected using Greenhouse–Geisser estimates of sphericity (ε = 0.70).

**Table 5:** Results of 2 × 12 ANOVA on the differences between RCU and MCU on motive subscales.

	ANOVA			
Factors	*F*	df	*p*	*η* ^2^
Motives	119.314	7.74	0.001	0.32
Cannabis user group	15.5	1	0.001	0.22
Motives*Cannabis User Group	8.219	7.74	0.001	0.20

RCU, Recreational Cannabis Users; MCU, Medical Cannabis Users.

Using the Greenhouse–Geisser correction, there was a significant main effect of motives [*F*(7.74, 1230.57) = 119.314, *p* < 0.001, *η*^2^ = 0.32], with *Enjoyment* (*M* = 11.08, *SD* = 3.55), *Low Risk* (*M* = 10.34, *SD* = 3.92), and *Sleep* (*M* = 9.66, *SD* = 4.14) motives having the highest overall scores and *Conformity* (*M* = 3.24, *SD* = 1.0) and *Alcohol* (*M* = 3.88, *SD* = 2.03) motives having the lowest overall scores regardless of group (Table 4; Figure 4).

There was a significant main effect of the cannabis user group *F*(1,159) = 15.5 *p* < 0.001, η^2^ = 0.22, with RCUs having a higher overall mean score on motive subscales (*M* = 91.10, *SD* = 2.39) than MCU (*M* = 76.80, *SD* = 2.46).

Using the Greenhouse–Geisser, there was a significant Motives*Cannabis User Group interaction *F*(7.74, 1230.57) = 8.219, *p* < 0.001, *η*^2^ = 0.20. Planned independent-sample *t*-tests were conducted to examine this interaction (Table 5). There were differences between the two groups on all the motive subscales apart from *Low Risk* and *Social Anxiety* (*p* > 0.05). RCUs scored higher on motive subscales of *Enjoyment, Coping, Experimentation, Boredom, Alcohol, Celebration, Altered Perception,* and *Availability* (*p* < 0.05). MCUs scored higher on the *Sleep* motive subscale (*p* = 0.017). All descriptive and *t*-test statistics and *p*-values are reported in Table 4.

## 4 Discussion

The primary aim of the current study was to investigate differences between RCUs and MCUs in the UK. The two main areas of interest were the differences in mental health (PTSD, depression, and anxiety) and motives for cannabis use. The current study also investigated other potential characteristics, including age, cannabis use frequency, and other substance use. The results showed differences in age, cannabis use frequency, state anxiety, and cannabis use motives between the two groups. MCUs were more likely to be older, present with a higher frequency of cannabis use, have higher scores on *Sleep* motive, and lower scores of state anxiety than RCUs. RCUs had higher scores on several motives, such as *Enjoyment, Conformity, Coping, Experimentation, Alcohol, Celebration, Altered Perception,* and *Availability* compared to MCUs. The current study found no significant differences between the two groups on PTSD, depression, trait anxiety, other substance use, and two motives (*Social Anxiety* and *Low Risk*).

### 4.1 Individual differences

Consistent with previous research (Choi et al., 2017; Camsari et al., 2019; Turna et al., 2020), the present study found that there were differences in age between the MCU and RCU groups, with MCUs being, on average, older than RCUs. These age-related differences could be a result of health-related disparities, with older adults being more likely to have symptoms that lead to them seeking CBMPs (McKee et al., 2021). Economic stability may be another factor in understanding these differences, as despite guidelines allowing for the use of CBMPs through the UK’s single-payer system, the National Health Service, for several conditions, most prescriptions are privately funded (Lusardi and Mitchell, 2011; FitzRoy and Nolan, 2020). This, in turn, could lead to younger people seeking treatment for their symptoms, relying on unregulated cannabis, which then leads to the potential for increased risk of harm from exposure to unregulated cannabis (Couch, 2020). Further research is needed to understand the mediating role economics has on risks associated with unregulated cannabis use.

Previous research has shown that RCUs are vulnerable to polydrug use (Lin et al., 2016). The present study did not display any differences in alcohol, tobacco, or caffeine use. However, as participants were not asked about illicit drug use, these findings do not suggest that RCUs have more prevalent use of alcohol, caffeine, or tobacco compared to MCUs. Consistent with previous research (Lin et al., 2016; Choi et al., 2017), MCUs were found to have a higher frequency of cannabis use compared with RCUs, which was expected given the regularity with which prescribed CBMPs must be taken.

### 4.2 Mental health differences

MCUs had a higher incidence of self-reported neurological problems, mood disorders, and anxiety disorders compared to RCUs. These differences were to be expected as the MCU group was recruited from a population who was already treating their symptoms with CBMPs. *State anxiety* scores were lower for the MCU group compared with the RCU group. These differences were statistically significant. Scores for depression (CES-D), PTSD (PCL-5), and trait anxiety (STAI) were slightly higher, trending toward significance for MCUs. Whilst MCUs are usually found to have elevated scores on mental health measures (Choi et al., 2017; Turna et al., 2020), it was unexpected to note the statistically different scores for state anxiety. This difference could be explained by their use of CBMPs, which has been shown to reduce anxiety in patient populations (Ergisi et al., 2022; Sachedina et al., 2022; Rifkin-Zybutz et al., 2023), thus providing further evidence for the anxiolytic properties of CBMPs, which have been increasing in prevalence since the onset of the COVID-19 pandemic (Shevlin et al., 2020a,b; Jenkins et al., 2021; Saeed et al., 2022).

### 4.3 Motives differences

A comparison of motives for cannabis use supported previous findings, indicating that subscales for *Enjoyment, Low Risk,* and *Sleep* showed the highest scores (Dekker et al., 2009; Gill et al., 2015; Blevins et al., 2016; Altman et al., 2019; Winiger et al., 2021). This was the case for both the RCU and MCU groups. *Low-Risk* motive was also high for both groups, which is supported by the abundance of research illustrating that cannabis is the most globally normalized illicit drug (Korf, 2006; Osborne and Fogel, 2007; Sznitman et al., 2013), with reports of its increasing popularization in recent years (Skliamis et al., 2021). In addition, legislative change has resulted in the legalization of recreational cannabis use in an increasing number of jurisdictions. MCUs were more likely to use cannabis to aid sleep. Haug et al. (2017) reported that middle-aged MCUs are more likely to use cannabis as a sleep aid and to cope with symptoms of insomnia. This result is consistent with our MCU cohort being older and the literature indicating that middle-aged adults have higher rates of insomnia than younger adults (Sepehrmanesh et al., 2010). Moreover, MCUs are affected by chronic health conditions which are likely to be affected by co-morbid sleep disorders, with data from the UK Medical Cannabis Registry suggesting that these individuals experience improvements in self-reported sleep quality after initiation of CBMPs (Olsson et al., 2023).

Contrary to previous literature, which suggests that MCUs often use cannabis to cope (Bonn-Miller et al., 2014), the present study indicates that RCUs are more likely to use cannabis for the *Coping* motive. To our knowledge, this is the first study directly comparing MCUs and RCUs’ motives for cannabis use. One explanation for these findings could be that both MCUs and RCUs score similarly on mental health measures, leading the RCU *Coping* motive to be high. For example, in individuals with attention-deficit/hyperactivity disorder, reported use of cannabis is higher than that among the general population (Francisco et al., 2022). However, it is anticipated that a large proportion of individuals with attention-deficit/hyperactivity disorder who remain underdiagnosed, particularly women (Quinn and Madhoo, 2014). Therefore, the consumption of cannabis in some RCUs may represent undiagnosed or sub-clinical psychiatric conditions, leading to an increase in the *Coping* motive. Future comparisons need to investigate differences in motives between the two cohorts and consider their potential association with mental health disorders.

### 4.4 Limitations

The findings of the current study should be considered in the context of their limitations and strengths. The main limitations include a relatively small sample size, self-reported assessments, and failure to control for the medical–recreational subgroup. Furthermore, the recruitment of participants from two different sources, a medical cannabis clinic for a patient population and social media from a recreational cannabis group, may also be a source of selection bias. This strategy, however, was utilized due to the differences in legality between medical and recreational cannabis use in the UK, with each strategy utilized to provide access to the most diverse sample possible. Future investigations should provide appropriate improvements to the current study design. Despite the limitations, the current findings have implications for future research, clinical practice, and legislation. Firstly, to our knowledge, this is the first study to investigate the potential differences in cannabis use motives between RCUs and MCUs. The current study provided considerable insight into the differences and overlaps in motives between the two groups, offering a substantial baseline for future comparisons. The current study is also the first to investigate differences between the two groups in the UK. The findings advocate the importance of conducting this type of comparison in countries other than Canada and the USA. The results showed different characteristics of cannabis users in this population, identified vulnerable groups for illicit cannabis use, and exemplified the potential consequences of the poor integration of CBMPs in the UK. These findings further support the need to develop a clear policy position on both medical and recreational cannabis use in the UK. It highlights two distinct populations that potentially require the development of separate legislation, whilst acknowledging there is also overlap between the motivations for cannabis use between the two groups. It is, therefore, important to develop further research into these two distinct yet overlapping populations with an aim of clarifying similarities and distinctions in more detail. Finally, these findings illustrate the need to investigate both the benefits of CBMPs, such as a reduction in symptoms of anxiety. Research in these areas could provide individuals with the knowledge they require making informed decisions when choosing to use or prescribe medical cannabis, which in turn could improve the integration of CBMPs in the UK, particularly in supporting developing and implementing policy.

## 5 Conclusion

Overall, the current study provides a foundation for future research investigating recreational and medical cannabis use in the UK. The findings propose clear differences between the two cohorts but also demonstrate areas of potential overlap. Notably, RCUs reported higher state anxiety at the time of the survey, despite similar levels of trait anxiety as MCUs. In addition, RCUs reported motives for cannabis use that were more closely associated with social reasoning, including *Enjoyment* or *Celebration*. Interestingly, they scored higher than MCUs on the *Coping* component of the CMMQ, which requires further examination in the future studies. MCUs were conversely older and reported a higher frequency of cannabis use. As the popularity of cannabis increases globally, and especially the use of CBMPs, this type of research will foster a more in-depth and substantial understanding of the risks and benefits associated with using cannabis recreationally and medically.

## Data availability statement

The raw data supporting the conclusions of this article will be made available by the authors, without undue reservation.

## Ethics statement

The studies involving humans were approved by the University of the West of Scotland School of Education and Social Sciences Ethics Committee. The studies were conducted in accordance with the local legislation and institutional requirements. The participants provided their written informed consent to participate in this study.

## Author contributions

BC: Conceptualization, Writing – original draft, Writing – review & editing, Writing – review & editing, Data curation, Formal Analysis, Investigation, Methodology. SE: Data curation, Writing – review & editing, Resources. MS: Resources, Writing – review & editing. LT: Conceptualization, Supervision, Writing – original draft, Writing – review & editing.
