# Corrigendum: Cannabis use in the UK: a quantitative comparison of individual differences in medical and recreational cannabis users

**DOI:** 10.3389/fpsyg.2024.1368554

**Published:** 2024-02-13

**Authors:** Beata Ciesluk, Simon Erridge, Mikael H. Sodergren, Lucy J. Troup

**Affiliations:** ^1^Division of Psychology, School of Education and Social Sciences, University of the West of Scotland, Paisley, United Kingdom; ^2^Medical Cannabis Research Group, Imperial College London, London, United Kingdom; ^3^Sapphire Medical Clinics, London, United Kingdom; ^4^Curaleaf International, London, United Kingdom

**Keywords:** cannabis, cannabinoid, medical cannabis, recreational drug use, anxiety, depression

In the published article, there were errors in the figure numbering and captions.

Figure 2 should be re-numbered to be Figure 5, and the caption should be changed to “Differences in mean scores on motive subscales between RCU and MCU”.

Figure 3 should be re-numbered to be Figure 2, with the caption “Differences in percentage frequency of self-reported current psychological diagnoses between RCU and MCU.”

Figure 4 should be re-numbered to be Figure 3, with the caption “Differences in mean score on mental health measures between RCU and MCU.”

Figure 5 should be re-numbered to be Figure 4, with the caption “Total sample mean scores on motives subscales.”

Additionally, in the published article, there were errors in the in-text figure citations.

Where Figure 3 has been cited in the text of Section 3.1.4 Current psychological diagnoses, this should instead be “Figure 2”. The corrected sentence is below.

“No differences between the two groups in substance use-related disorders and other psychological disorders were found (*p* > 0.050) (see Figure 2; Table 1).”

Where Figure 4 has been cited in the text of Section 3.2 Mental health, this should instead be “Figure 3”. The corrected sentence is below.

“This interaction is illustrated in Figure 3.”

Where Figure 4 has been cited in Section 3.3 Motives, this should instead be “Figure 5”. The corrected sentence is below.

“Using the Greenhouse–Geisser correction, there was a significant main effect of motives [*F*_(7.74, 1230.57)_ = 119.314, *p* < 0.001, η^2^ = 0.32], with *Enjoyment* (*M* = 11.08, *SD* = 3.55), *Low Risk* (*M* = 10.34, *SD* = 3.92), and *Sleep* (*M* = 9.66, *SD* = 4.14) motives having the highest overall scores and *Conformity* (*M* = 3.24, *SD* = 1.0) and *Alcohol* (*M* = 3.88, *SD* = 2.03) motives having the lowest overall scores regardless of group (Table 4; Figure 5).”

Where Figure 5 has been cited in the text of Section 3.2 Mental health, this should instead be “Figure 3”. The corrected sentence is below.

“As observed in Figure 3, RCUs scored lower on all the mental health measures except from *State Anxiety* scores (*p* < 0.001).”

Additionally, in Section 3.1.3 Substance use, instead of “(see Figure 2; Table 1)”, only Table 1 should be cited here. A correction has been made to **Section 3 Results**, “*3.1.3 Substance use*”. The corrected paragraph is shown below.

“Considering substance use prior to completing the survey, there were only significant differences for cannabis use (*p* = 0.006) (see Table 1), with MCUs presenting a higher frequency of cannabis use 24 h prior to taking the survey (*n* = 71; 88.75%) than RCUs (*n* = 53; 66.25%; *p* < 0.001) and 8 h prior to completing the survey (*n* = 49; 61.25%; *p* = 0.006) than RCUs (*n* = 32; 40%; *p* = 0.006). There were no significant differences in caffeine, alcohol, and tobacco use between the two groups (*p* > 0.050).”

Lastly, in Section *3.3 Motives*, Figure 4 and Figure 5 should be cited along with Table 4 and Table 5, respectively. Corrections have been made to **Section 3 Results**, “*3.3 Motives*”, Paragraphs 1 and 2. The corrected paragraphs are shown below.

“The differences in motives for cannabis use between RCUs and MCUs were assessed by the CMMQ, which has 12 subscales (*Enjoyment, Conformity, Coping, Experimentation, Boredom, Alcohol, Celebration, Altered Perception, Social Anxiety, Low Risk, Sleep, and Availability*). Descriptive statistics for each subscale and group scores are displayed in Table 4 and Figure 4.”

“The differences in motives between MCUs and RCUs were analyzed using a mixed-design (2 × 12) ANOVA (see Table 5; Figure 5) with within-subject factors of motives subscales (*Enjoyment, Conformity, Coping, Experimentation, Boredom, Alcohol, Celebration, Altered Perception, Social Anxiety, Low Risk, Sleep, and Availability*) and between-subject factors of cannabis user group (RCUs and MCUs). Mauchly's test of sphericity indicated that the assumption of sphericity had been violated [$\chi_{\left(65\right)}^{2}$ = 0.084, *p* < 0.001]; therefore, degrees of freedom were corrected using Greenhouse–Geisser estimates of sphericity (ε = 0.70).”

The authors apologize for these errors and state that they do not change the scientific conclusions of the article in any way. The original article has been updated.

